# Density-dependent dispersal reduces conflict over the sex ratio

**DOI:** 10.1098/rsbl.2024.0378

**Published:** 2024-10-30

**Authors:** Chedhawat Chokechaipaisarn, Andy Gardner

**Affiliations:** ^1^ School of Biology, University of St Andrews, St Andrews, Fife KY16 9TH, UK

**Keywords:** sex ratio conflict, density dependence, sex allocation, viscosity, haplodiploidy, constant non-disperser principle

## Abstract

Haplodiploids—in particular, wasps—are the workhorses of sex-allocation research. This is owing to their unusual system of sex determination, which provides a ready means of sex ratio adjustment. Notably, their sexually asymmetrical mode of genetic inheritance leads mothers and fathers to come into conflict over the sex ratio of their offspring. In the simplest outbreeding scenario, a mother is favoured to employ an even sex ratio while a father prefers that all his mate’s offspring are female. An important modulator of evolutionary conflict between mating partners is genetic relatedness, raising the possibility that this sex ratio conflict is reduced in low-dispersal settings with mating occurring between relatives. However, the impact of population viscosity on sex ratio conflict in haplodiploids remains unknown. Here, we develop and analyse a kin-selection model to investigate how the rate of dispersal modulates sex ratio conflict in a haplodiploid, viscous population setting. We find that population viscosity is associated with a reduction in the extent of sex ratio conflict—the effect being very weak under density-independent dispersal and much stronger under density-dependent dispersal.

## 1. Introduction

The study of sex allocation provides among the best quantitative evidence for the power and precision of Darwinian adaptation [1]. Haplodiploids, e.g. wasps, represent around 15% of all arthropod species and are the workhorses of sex-allocation research owing to their unusual system of sex determination, which provides a ready means of sex ratio adjustment [1–4]. Haplodiploidy in its standard form involves diploid daughters being produced sexually, with one genome being contributed from their mother and one genome being contributed from their father, and haploid males being produced asexually from unfertilized eggs. This sexually asymmetrical mode of genetic inheritance means that mothers and fathers may come into conflict over the sex ratio of their offspring; in a panmixia scenario, mothers are favoured to adopt an even sex ratio whereas fathers are favoured to have all their mates’ offspring be female [5].

An important modulator of evolutionary conflict between mating partners is their genetic relatedness—i.e. the degree of inbreeding [6–8]. Consequently, one might expect this conflict over the sex ratio to be reduced in viscous populations, whereby a low rate of individual dispersal leads to high relatedness between mating partners. However, a classic result states that—in a simple viscous population setting—a mother’s optimal sex ratio is essentially invariant with respect to dispersal rate [9–12]. This invariant result has been generalized to encompass other social behaviours, such as altruism [13], and has received a huge amount of attention in the study of social evolution (reviewed by Taborsky *et al*. [14]). Moreover, the effect—if any—of the rate of dispersal upon a father’s optimal sex ratio under haplodiploidy does not appear to have been investigated. Accordingly, the impact of population viscosity on sex ratio conflict in haplodiploids remains obscure.

Here, we develop and analyse a kin-selection model to investigate how the rate and pattern of dispersal modulates sex ratio conflict in a haplodiploid, viscous population setting. First, we determine a father’s optimal sex ratio as a function of the rate of density-independent dispersal. Second, we investigate the impact of the rate of density-dependent dispersal on a father’s optimal sex ratio. Third, we contrast these paternal sex ratio optima with previous results concerning maternal sex ratio optima, quantifying the degree of sex ratio conflict as a function of the rate and pattern of dispersal. We find that population viscosity is associated with a reduction in the extent of sex ratio conflict—the effect being very weak under density-independent dispersal and much stronger under density-dependent dispersal.

## 2. Results

### (a) Mathematical model

We assume an infinite haplodiploid population separated into patches, with each patch being founded by *n* fertilized females, and each of these mothers producing a large number of offspring of both sexes, with offspring of each sex being equally costly to produce. All mothers die after reproduction, and upon reaching adulthood, the offspring mate at random within their patch, with each female mating once and each male mating potentially multiple times. Following the mating phase, all males die, and mated daughters either remain in their natal patch or else attempt to disperse to new, randomly chosen patches. A proportion *c* of dispersing females are killed *en route*, and a proportion 1 *− c* survive to compete for breeding opportunities in their new patches. Following dispersal, *n* mated females are chosen at random on each patch to be the mothers of the next generation, with all other females perishing, and this returns the population to the beginning of the life cycle.

### (b) Density-independent dispersal

We begin by assuming that all mated females attempt to disperse with a constant probability *d*. Previous work has shown that in this context a mother’s optimal sex-allocation strategy—i.e. the proportion of her offspring that are male—is given by [11,15]


(2.1) $$z^{*}=\;\frac{n-1}{2n}\;\frac{4n\;-\;\left(n-1\right){\left(\left(1-d\right)/\left(1-dc\right)\right)}^{2}-\;2}{4n\;-\;\left(n-1\right){\left(\left(1-d\right)/\left(1-dc\right)\right)}^{2}-\;1}.$$

**Figure 1.**
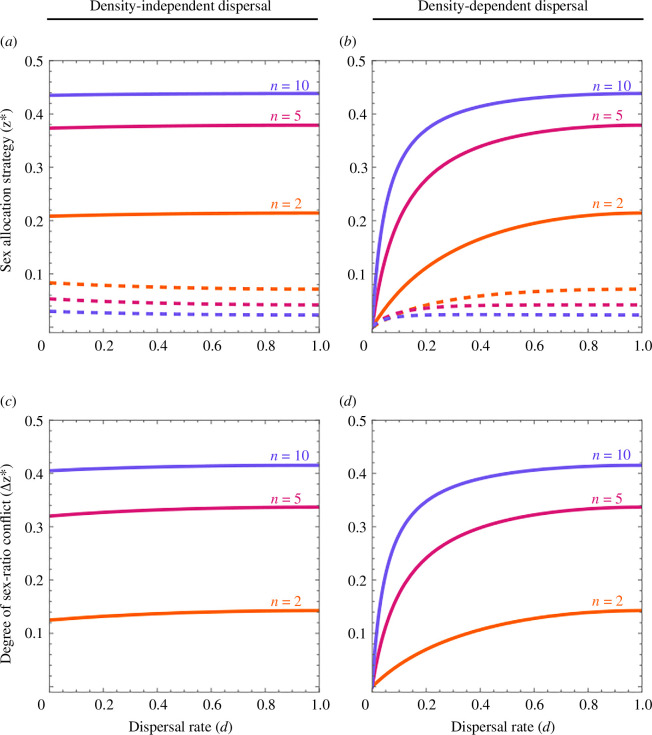
Optimal sex allocation strategy (z*) from the perspective of a mother (solid line) and a father (dashed line) under (*a*) density-independent dispersal and (*b*) density-dependent dispersal, as a function of the overall rate of dispersal (*d*) and with costless dispersal (*c* = 0). The absolute difference between the mother’s and father’s optimal sex-allocation strategies defines the degree of sex ratio conflict (Δz*), shown here under (*c*) density-independent dispersal and (*d*) density-dependent dispersal, as a function of the overall rate of dispersal (*d*) and with costless dispersal (*c* = 0). Shown are the results for two (*n* = 2, orange), five (*n* = 5, red) and ten (*n* = 10, purple) foundresses.

A smaller number of foundresses (i.e. lower *n*) is associated with a higher level of relatedness within the mating group, and hence she is favoured to exhibit a more female-biased sex ratio as this enhances the overall productivity of the mating group (figure 1*a*, solid lines). In the extreme of single-foundress patches (i.e. *n* = 1), and hence genetically identical mating partners, her optimal sex ratio is close to zero—i.e. to produce just as many sons as are required to ensure her daughters are mated [5]. However, although a lower rate of dispersal (i.e. lower *d*) is also associated with a higher level of relatedness, it has negligible impact upon the mother’s optimal sex-allocation strategy (figure 1*a*, solid lines). This owes to lower dispersal also being associated with an intensification of resource competition between genetically related females, which reduces the adaptive value of daughters [9–11].

Using kin-selection methodology [16,17], we find that a father’s optimal sex-allocation strategy is instead given by (see electronic supplementary material for details)


(2.2) $$z^{*}=\;\frac{n-1}{n\left(4n-\left(n-1\right){\left(\left(1-d\right)/\left(1-dc\right)\right)}^{2}-1\right)}.$$

As he contributes genes to his mates’ daughters, but not to their sons, he is favoured to exhibit a strongly female-biased sex ratio (figure 1*a*, dashed lines). Note that, on account of his being somewhat genetically related to his mates, he will tend to have some degree of relatedness to his mates’ sons, and hence they are not completely devoid of adaptive value from his perspective [18]. Accordingly, for all multi-foundress (i.e. *n* > 1) scenarios, a smaller number of foundresses, which is associated with higher relatedness within the mating group, leads to him being favoured to exhibit a less female-biased sex ratio. In the exceptional case of a single foundress (*n* = 1) in each patch, chronic inbreeding ensures all offspring are genetically identical—aside from *de novo* mutation—and here the father’s sex ratio interests exactly coincide with the mother’s (i.e. z* = 0). Also, in the special case of full dispersal (*d* = 1), equation (2.2) reduces to Taylor & Bulmer’s [19] result z* = (*n*− 1)/(*n*(4*n* − 1)). A lower rate of dispersal (i.e. lower *d*) is associated with higher relatedness within the mating group, which reduces the extent to which he favours a female-biased sex ratio, but this is—once again—a weak effect (figure 1*a*, dashed lines).

### (c) Density-dependent dispersal

We now consider that mated females are able to adjust their probability of dispersal according to the number of mated females in their patch prior to dispersal (local female density). Specifically, we suppose that Crespi & Taylor’s [20] ‘constant non-disperser’ (CND) principle applies—as was shown by Chokechaipaisarn & Gardner [15] in this same population context. That is, a female’s probability of dispersal is given by


(2.3) $$d_{D}=1-\frac{1-d}{D},$$

where *d* is the overall proportion of females dispersing across the whole population, and *D* is the ratio of local female density and the average number of mated females per patch across the whole population [15]. This means that the absolute number of non-dispersing females in any patch is proportional to (1 − *d_D_*) × *D* = 1 − *d*, i.e. it is the same for all patches, irrespective of their densities [20].

In this density-dependent dispersal context, we find that a mother’s optimal sex-allocation strategy is given by (see electronic supplementary material for details)


(2.4) $$z^{*}=\;\frac{\left(n-1\right)\left\{2\left(2n-1\right)-\left(n-1\right){\left(\left(1-d\right)/\left(1-dc\right)\right)}^{2}\right\}}{2\{\left(n-1\right)-(\left(1-dc\right)/({\left(1-d\right)}^{2}-(1-d^{2}c)))\}\;\{4n-\left(n-1\right)(\left(1-d\right)/\left(1-dc\right){)}^{2}-1\}}.$$

Note that the optimal overall rate of dispersal is d* = 2/(1 + 2*cn *+ (1 + 4*n*(*n *− 1)*c*^2^)^1/2^) [21–24], and substituting this into equation (2.4) recovers equation (2.6) of Chokechaipaisarn & Gardner [15]. Here, density dependence decouples the relatedness and kin-competition consequences of dispersal [25], such that a lower overall rate of dispersal is associated with higher relatedness within mating groups but does not incur exacerbated competition for resources among female relatives, such that a mother is favoured to exhibit a more female-biased sex ratio (figure 1*b*, solid lines; [15]).

In this same density-dependent dispersal context, we find that a father’s optimal sex-allocation strategy is given by (see electronic supplementary material for details)


(2.5) $$z^{*}=\;\frac{\left(n-1\right)}{\left\{\left(n-1\right)-(\left(1-dc\right)/({\left(1-d\right)}^{2}-(1-d^{2}c)))\}\;\{4n-\left(n-1\right){\left(\left(1-d\right)/\left(1-dc\right)\right)}^{2}-1\right\}}.$$

As in the density-independent dispersal scenario, a father’s sex ratio optimum is highly female biased, with a higher proportion of daughters being favoured as the foundress number increases (figure 1*b*, dashed lines). However, in contrast to the density-independent dispersal scenario, in which fathers favour a less female-biased sex ratio as the rate of dispersal increases (figure 1*a*, dashed lines), in this density-dependent dispersal context, fathers favour a more female-biased sex ratio as the rate of dispersal increases—albeit weakly (figure 1*b*, dashed lines).

### (d) Sex ratio conflict between mates

Consideration of the sex-allocation interests of mothers and fathers has revealed that they are usually in conflict, for both density-independent and density-dependent dispersal scenarios. Here, we investigate how the rate and pattern of dispersal impact upon the extent of sex ratio conflict. Under density-independent dispersal, the degree of sex ratio conflict may be expressed as


(2.6) $$\Delta z^{*}=\;\frac{{\left(n-1\right)}^{2}}{2n}\left[\frac{4-{\left(\left(1-d\right)/\left(1-dc\right)\right)}^{2}}{4n\;-\;\left(n-1\right){\left(\left(1-d\right)/\left(1-dc\right)\right)}^{2}-\;1}\right],$$

which is obtained by subtracting the father’s optimum (equation 2.2) from the mother’s (equation 2.1) for the density-independent dispersal scenario. The degree of sex ratio conflict decreases as the rate of density-independent dispersal decreases—but only very weakly (figure 1*c*).

Under density-dependent dispersal, the degree of sex ratio conflict is


(2.7) $$\Delta z^{*}=\;\frac{{\left(n-1\right)}^{2}}{2}\left[\frac{4-{\left(\left(1-d\right)/\left(1-dc\right)\right)}^{2}}{(4n-\left(n-1\right){\left(\left(1-d\right)/\left(1-dc\right)\right)}^{2}-1)\left\{\left(n-1\right)-(\left(1-dc\right)/({\left(1-d\right)}^{2}-(1-d^{2}c)))\right\}}\right],$$

which is obtained by subtracting the father’s optimum (equation 2.5) from the mother’s (equation 2.4) for the density-dependent dispersal scenario. Here, the degree of sex ratio conflict also decreases as the rate of density-dependent dispersal decreases—though much more dramatically—and is extinguished altogether in the limit of a fully viscous population (figure 1*d*). When the overall rate of dispersal is at its optimum (i.e. d*), a lower rate of density-independent or density-dependent dispersal (i.e. owing to a higher cost of dispersal, *c*) leads to a reduction in the extent of sex ratio conflict, with the extent of sex ratio conflict under density-dependent dispersal being lower than that under density-independent dispersal for all non-zero costs of dispersal (0 < *c* ≤ 1) and assuming multiple-foundress patches (*n *> 1; see electronic supplementary material for details). That is, population viscosity serves as a mechanism for reducing the extent of sex ratio conflict, and its effect is much stronger at lower rates of density-dependent dispersal.

## 3. Discussion

We have investigated the impact of the rate and pattern of dispersal on the extent of sex ratio conflict between mating partners under a haplodiploid mode of genetic inheritance. Previously, a mother’s sex ratio optimum has been shown to be approximately invariant with respect to the rate of density-independent dispersal [11] and strongly modulated by the rate of density-dependent dispersal [15], with a more female-biased sex ratio being favoured in a more viscous population setting. Here, we have shown that a father’s sex ratio optimum is also approximately invariant with respect to the rate of density-independent dispersal and is strongly modulated by the rate of density-dependent dispersal, with a more female-biased sex ratio being favoured in a more viscous population setting. Consequently, we have found that the extent of sex ratio conflict between mating partners is ameliorated by population viscosity, only very weakly under density-independent dispersal and much more strongly under density-dependent dispersal.

A reduced rate of density-independent dispersal leads not only to higher relatedness but also to more resource competition between female kin [10]. This leads to a mother’s optimal sex ratio being exactly invariant with respect to the rate of density-independent dispersal under haploidy and diploidy [9,10,12] and approximately invariant under haplodiploidy [11]—there being a small additional bias towards females in a more viscous population setting under haplodiploidy owing to inbredness leading mothers to be relatively more consanguineous with their daughters [26,27]. We have shown that a similar relationship obtains when taking a father’s perspective, with the opposing effects of relatedness and resource competition leading to his optimal sex ratio being exactly invariant with respect to the rate of density-independent dispersal under haploidy and diploidy (see electronic supplementary material for details) and approximately invariant under haplodiploidy—there being a slightly reduced bias towards females in a more viscous population setting owing to inbredness leading males to be relatively more consanguineous with their mates’ sons. Consequently, there is a weak tendency for a reduced rate of density-independent dispersal to decrease the extent of sex ratio conflict between mating partners in haplodiploid populations.

If individuals are able to adjust their probability of dispersing according to local density, then they are favoured to do so [20], and this results in a decoupling of the relatedness and resource competition effects [25]. This leads to a mother’s optimal sex ratio being strongly dependent on the rate of density-dependent dispersal, with a strongly increasing bias towards females in a more viscous population setting, for haploid, diploid and haplodiploid modes of genetic inheritance [15]. We have shown that a similar relationship is obtained when taking a father’s perspective, with an increasingly female-biased sex ratio being favoured in a more viscous population setting, under haploidy, diploidy and haplodiploidy, on account of density-dependent dispersal alleviating resource competition among female relatives. This results in a situation whereby a reduced rate of density-dependent dispersal leads to an alignment of mating partners’ sex ratio optima, such that the extent of sex ratio conflict is greatly diminished, in haplodiploid populations.

To facilitate our analysis, we have made several simplifying assumptions, and relaxing these may represent a useful avenue for further theoretical analysis. We have assumed that females and males are equally costly to produce, and although relaxing this assumption is not expected to affect our results concerning sex allocation—i.e. the proportion of parental resources invested into the production of sons versus daughters—it will affect the resulting sex ratio—i.e. the proportion of offspring that are male. We have also assumed that females exhibit monogamous mating; female promiscuity is known to modulate parent–offspring conflict over sex allocation in haplodiploids [28], though its impact on sexual conflict over the sex ratio in viscous populations remains to be investigated. We have assumed that male fecundity is not limiting, such that even in the limit of their being vanishingly rare, there are still sufficient males to ensure that all females are successfully mated. The consequences of male limitation in the context of local mate competition have been studied for haploidy and diploidy [29–32] but remain to be determined for haplodiploidy. We have also assumed that there is no kin recognition, and relaxing this assumption could have both a direct impact on sex-allocation behaviour [33,34] as well as an indirect impact via inbreeding avoidance behaviour [35].

We have taken a ‘battleground’ approach [36] to determine the extent of sex ratio conflict—giving first the mother and then the father full control over the sex ratio of their offspring—and quantifying the difference between the resulting sex ratio optima. More generally, parents are expected to share control over the sex ratio, with the typical assumption being that mothers have more control than fathers in species where males die after mating and are only able to exert their influence via their sperm. A ‘resolution’ approach [36]—that explicitly models shared parental control over the sex ratio and incorporates details such as the temporal ordering of their decisions—may yield further insights into how the sex ratio conflict plays out [37,38]. In addition, we have assumed that female dispersal occurs after mating; the timing of dispersal relative to mating has been shown to modulate sexual conflicts in viscous populations [7,39]. We have also assumed that the destination patch is decided independently for each disperser. Budding dispersal—whereby patchmates travel together to a shared destination—is understood to modulate sex-allocation behaviour [12], but its impact on sex ratio conflict remains to be investigated. Finally, sex ratio conflict between mating partners might be modulated by the presence of non-reproductive individuals, such as helpers [40] and soldiers [39].

Empirical research on sex allocation has overwhelmingly focused on the fitness interests of the mother [41]. However, the role of the father to exert influence over the sex ratio is increasingly receiving attention [42–44]. Paternal influence on the sex ratio has been shown in a number of taxa including parasitoid wasps [45,46], spider mites [47], spiders [48] and mammals [42,43], and involving a range of mechanisms [49]. Haplodiploid spider mites might provide an excellent opportunity for testing the present theoretical predictions on account of their amenability to experimental evolution investigation [47,50,51]. More generally, owing to the equivalence between haplodiploid and X-chromosomal modes of genetic inheritance, the predictions of the present analysis might also be applied to diploid taxa in which one or both parents’ influence over the sex ratio has an X-linked genetic basis.

## Data Availability

This article has no additional data. Supplementary material is available online [52].
